# Investigation of spin-orbit torque using current-induced magnetization curve

**DOI:** 10.1038/s41598-017-00962-7

**Published:** 2017-04-11

**Authors:** Tomohiro Koyama, Yicheng Guan, Daichi Chiba

**Affiliations:** Department of Applied Physics, The University of Tokyo, Bunkyo, Tokyo 113-8656 Japan

## Abstract

Manipulation of magnetization using current-induced torque is crucial for magnetic recording devices. Recently, the spin-orbit torque (SOT) that emerges in a ferromagnetic thin film on a heavy metal is focused as a new scheme for magnetization switching in perpendicularly magnetized systems. Since the SOT provides a perpendicular effective field to the system, the formation of a magnetic multiple domain state because of Joule heating is supressed in the magnetization reversal process. This means that high reliable switching is possible using the SOT. Here, by utilizing the SOT induced domain stability, we show that an electrical current directly injected to a perpendicularly magnetized Pt/Co/Pd system can magnetize itself, that is, current-induced magnetization process from multi to single domain state. A quantitative determination of the SOT is performed using the current-induced magnetization curve. The present results are of great importance as another approach to evaluate the SOT effect, as well as a demonstration of domain state switching caused by the SOT.

## Introduction

In magnetic recording devices, the information of a bit is retained as a magnetization direction. Spin-torque-induced magnetization switching^1–6^ in magnetic tunnel junctions (MTJs) and current-induced domain wall (DW) displacement^7–13^ in magnetic wires have been widely investigated as information writing methods in magnetic memory. Recently, a current-induced spin-orbit torque (SOT) emerging in a thin ferromagnetic film deposited on a heavy metal layer has been recognized as a new scheme for magnetization switching in perpendicularly magnetized materials^14–19^. Moreover, it is well known that current-induced DW dynamics is strongly affected by the SOT^20–24^.

**Figure 1:**
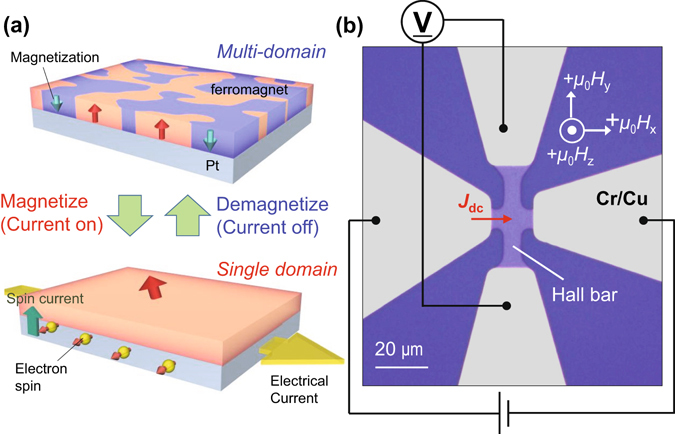
Current-induced magnetization process and schematic of device structure. (**a**) When electrical current is injected into thin ferromagnet/Pt layered structure, the system is gradually magnetized and finally, single magnetic domain structure appears. (**b**) Device image taken by an optical microscope. A Pt/Co/Pd Hall bar structure with 5 μm width is prepared. Grey regions indicate Cr/Cu electrodes. Current source and voltage meter are connected as shown in the figure. Directions of external magnetic fields (*μ*_0_ *H*_*x*_, *μ*_0_ *H*_*y*_ and *μ*_0_ *H*_*z*_) are indicated by white arrows. The sign of electrical current is defined as positive when the current flows from left to right.

The SOT is known to act on magnetizations as an effective field. This contributes to the magnetization switching and DW motion. More importantly, the SOT effective field also plays a role in stabilizing the domain state against current-induced Joule heating^25, 26^, which is often a severe problem in devices based on current-induced torque^27–29^. Owing to this characteristic of the SOT, random multiple domain formation is suppressed even under high current density as much as 10^11^–10^12^ A/m^2^ during the magnetization reversal. In this letter, we show that in a perpendicularly magnetized Pt/Co/Pd structure with a multi domain (MD) state in thermal equilibrium (having no net magnetization), the magnetization of the system increases with injected current, and finally, a single domain (SD) state is created (see Fig. 1a). This “current-induced” magnetization process is observed only when an external in-plane magnetic field parallel to the current exists, indicating that the SOT is responsible for the effect. A quantitative determination of the SOT effective field from the current-induced magnetization curve is performed.

Figure 1b shows an optical microscope image of our device. The Hall bar component consists of asymmetrical Pt/Co/Pd layers deposited on a SiO_2_/intrinsic Si substrate (see also Methods). The width of the channel is 5 μm. Owing to the interface magnetic anisotropy at the Pt/Co and Co/Pd interfaces, the system exhibits perpendicular magnetic anisotropy PMA. The high PMA realizes an MD state with perpendicular magnetization near the Curie temperature (~370 K in our device). Four Cr/Cu electrodes are formed to apply the current and to detect the Hall resistance *R*_Hall_. The definitions of each external magnetic field (*μ*_0_ *H*_*x*_, *μ*_0_ *H*_*y*_, and *μ*_0_ *H*_*z*_) and the current flow along the *x*-axis are indicated in Fig. 1b. The temperature of the stage *T*_s_, which is in thermal contact with the fabricated device, is controlled using a heater (see Methods).

**Figure 2:**
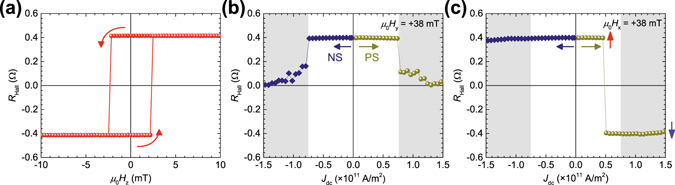
Anomalous Hall measurement under various dc currents. (**a**) Results of anomalous Hall resistance *R*_Hall_ measurement by sweeping perpendicular field *μ*_0_ *H*_*z*_. Densities of dc current *J*_dc_ used here are +2.8 × 10^9^ A/m^2^. Sweep rate of *μ*_0_ *H*_z_ was ~0.04 mT/s. Measurements were performed at stage temperature of 304 K. Red arrows indicate the sweep direction. (**b**,**c**) *R*_Hall_ as a function of *J*_dc_ obtained under *μ*_0_ *H*_y_ (**b**) and *μ*_0_ *H*_x_ (**c**) of +38 mT. Shaded area indicates the *J*_dc_ region where multiple domain state appears under *y*-field. The error bar, which is the standard deviation of two data points, is smaller than the symbols.

Figure 2a shows the results of the *R*_Hall_ measurement when a dc current density *J*_dc_ of +2.8 × 10^9^ A/m^2^ is injected at room temperature (*T*_s_ = 304 K). *μ*_0_ *H*_*z*_ is swept to obtain the curves. *J*_dc_ is determined by simply dividing the current by the cross-sectional area of the metallic layers. *R*_Hall_ is proportional to the perpendicular component of the magnetization because of the anomalous Hall effect. A clear hysteresis loop with a coercivity *μ*_0_ *H*_c_ of 2.4 mT is observed. The remanent value of *R*_Hall_ (*R*_Hall_^r^) is almost equal to the saturation value, indicating that the SD state is stable at fields near zero.

Next, *R*_Hall_ measurements with *J*_dc_ sweeping are performed under constant in-plane magnetic fields. The procedure is as follows. First, the magnetization direction of the entire device is set upward by applying a *μ*_0_ *H*_*z*_ of +30 mT. After returning to ~0 T, a constant in-plane magnetic field (*μ*_0_ *H*_*x*_ or *μ*_0_ *H*_*y*_) of +38 mT is applied. Then, *R*_Hall_ is monitored with a sweeping *J*_dc_. The positive (negative) sweep corresponds to a *J*_dc_ change from 0 to +1.7 (−1.7) × 10^11^ A/m^2^. Figure 2b and c show *R*_Hall_ as a function of *J*_dc_ measured under the application of *μ*_0_ *H*_*y*_ and *μ*_0_ *H*_*x*_, respectively. As shown in the positive sweep (PS) in Fig. 2b, *R*_Hall_ abruptly decreases when a *J*_dc_ of +0.8 × 10^11^ A/m^2^ is applied, and drops toward zero in the region of *J*_dc_ > +0.8 × 10^11^ A/m^2^, indicating that the MD state is formed at *J*_dc_ ≥ 0.8 × 10^11^ A/m^2^ due to the Joule heating. A rapid decrease in *R*_Hall_ also appears symmetrically in the negative sweep (NS) case (see NS curve in Fig. 2b), suggesting that the Joule heating effect is independent of the *J*_dc_ direction.

By contrast, the situation is completely different when *μ*_0_ *H*_*x*_, which is parallel to *J*_dc_, is applied. For the PS curve in Fig. 2c, a full *R*_Hall_ switching from positive to negative, which corresponds to the abrupt reversal of the magnetization direction from up to down, is observed at *J*_dc_ = +0.5 × 10^11^ A/m^2^. In the NS curve, no switching occurs up to *J*_dc_ = −1.7 × 10^11^ A/m^2^. When the sign of *μ*_0_ *H*_*x*_ is reversed, switching is observed only for a negative *J*_dc_ (not shown). The switching direction in the present configuration is consistent with the SOT induced switching previously reported in the Pt/Co structure^2, 26^. Although the sign of the spin Hall angle of the top Pd is the same as that of the Pt, the SOT in the present device is dominated by the spin current injection from the bottom Pt because the magnitude of the spin Hall angle of Pd is one order smaller than that of Pt^30, 31^. We also checked that in a similar device structure, the top Pd effect on the SOT is negligibly small^32^. In order to evaluate the magnitude of SOT effective fields, *i.e*. the Slonczewski-like torque *μ*_0_ *H*_SL_ and field-like torque *μ*_0_ *H*_FL_, the harmonic Hall measurement, which is widely used for the quantitative determination of the SOT^33^, was conducted for a similar device structure. The procedure is shown in the Supplementary Information. *μ*_0_ *H*_SL_ and *μ*_0_ *H*_FL_ are determined to be 6.55 ± 0.20 mT/10^11^ A/m^2^ and 1.57 ± 0.13 mT/10^11^ A/m^2^, respectively. These values are close to those previously reported for Pt/ferromagnet bilayer structure^22^. Another important point here is that under *μ*_0_ *H*_*x*_ application, |*R*_Hall_| always represents a value close to saturation even at |*J*_dc_| ≥ 0.8 × 10^11^ A/m^2^, indicating that the MD formation is completely suppressed against Joule heating. In the present case, the magnetization experiences a finite perpendicular effective field *μ*_0_ *H*_eff_ derived from *μ*_0_ *H*_SL_ because the magnetization tilts slightly toward the *x*-direction. When the current direction is the same as the *μ*_0_ *H*_*x*_ direction, the sign of *μ*_0_ *H*_eff_ becomes negative in the Pt/Co system. As a result, the SD state becomes stable because of the gain by the Zeeman energy reduction. Thus, the SOT plays an important role in stabilizing the SD state.

**Figure 3:**
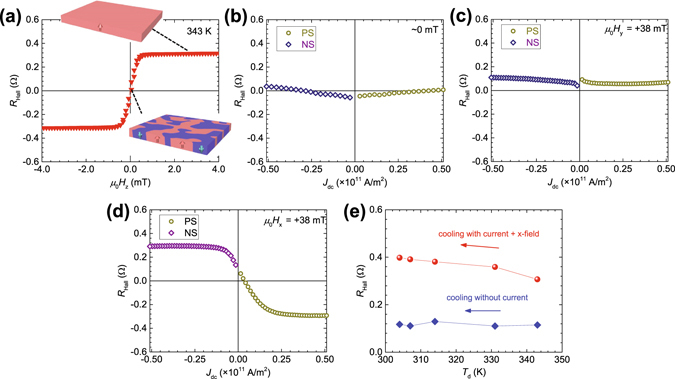
Current sweep measurement under 343 K. (**a**) *μ*_0_ *H*_z_ – *R*_Hall_ curve obtained at stage temperature *T*_d_ of 343 K. *J*_dc_ of +2.8 × 10^9^ A/m^2^ is used for the measurement. (**b–d**) *J*_dc_ dependences of *R*_Hall_ measured under (**b**) 0 T, (**c**) *μ*_0_ *H*_y_, and (**d**) *μ*_0_ *H*_x_ of +38 mT, respectively. Yellow (purple) points indicate results for the positive (negative) current sweep. (**e**) Results of *T*_d_ cooling experiment. *T*_d_ is reduced from 343 K to 304 K with injecting *J*_dc_ of −0.25 × 10^11^ (red) and −2.8 × 10^9^ A/m^2^ (blue) under *μ*_0_ *H*_x_ = +38 mT. The error bar, which is the standard deviation of two data points, is smaller than the symbols.

In the above experiments, *T*_d_ was increased by injecting a current. In the following, the MD state at thermal equilibrium is prepared by simply increasing *T*_d_ using a heater. Figure 3a shows the results of the Hall measurements performed at *T*_s_ = 343 K. *J*_dc_ for this measurement is 2.8 × 10^9^ A/m^2^, and the Joule heating effect is negligibly small. In this case, an anhysteric *R*_Hall_ curve with the almost zero remanent is observed. This indicates that the MD state is realized and the system is demagnetized. Figure 3b shows *R*_Hall_ as a function of *J*_dc_ obtained under *μ*_0_ *H*_*z*_ of ~0 T. *R*_Hall_ is almost independent of *J*_dc_ in the range of ±0.5 × 10^11^ A/m^2^. Similarly, no change in *R*_Hall_ is observed for a *μ*_0_ *H*_*y*_ of +38 mT, as shown in Fig. 3c. The slight *R*_Hall_ deviation from zero is probably due to the small *z*-component of the field. The slight *J*_dc_ dependence on *R*_Hall_ shown in Fig. 3b and c might be a result of the Oersted field. Based on these results, it can be concluded that in both cases, the MD state is kept under current injection. The result obtained under *μ*_0_ *H*_*x*_ = +38 mT is completely different, as shown in Fig. 3d. For a small *J*_dc_, *R*_Hall_ showed an intermediate value, indicating that the MD state remains. However, a clear increase and decrease in *R*_Hall_ with increasing current toward the negative and positive magnetization directions, respectively, are observed. For both current directions, *R*_Hall_ saturates above |*J*_dc_| = 0.1–0.2 × 10^11^ A/m^2^ and the *R*_Hall_ saturation values are consistent with those for the SD state. This result indicates that magnetization process of the system is caused by the SOT.

In order to check whether the device is fully in the SD state, a cooling experiment under current is carried out. The procedure is as follows. After making *R*_Hall_ saturation using current under *μ*_0_ *H*_*x*_ = +38 mT, *T*_d_ is gradually decreased from 343 K to room temperature with remaining the current and *x*-field application. *J*_dc_ of −0.25 × 10^11^ A/m^2^ is continuously applied during the cooling. During the *T*_d_ decrease, *R*_Hall_ is monitored. The result is shown in Fig. 3e. One can see that *R*_Hall_ gradually increases with decreasing *T*_d_ and at room temperature it shows ~0.4 Ω, which is the value for the SD state. This indicates that the SD state is created by the current at 343 K and is maintained during cooling. On the other hand, when the above experiment is performed with an injection of *J*_dc_ = −2.8 × 10^9^ A/m^2^, *R*_Hall_ shows a small value of ~0.1 Ω even at room temperature, suggesting that the MD state formed at 343 K is retained even at room temperature. Therefore, the *R*_Hall_ saturation in Fig. 3(d) corresponds to the complete SD state, *i.e*. a situation where no domain with an opposite magnetization exists in the device is realized against thermal agitation. The result presented here demonstrates that the magnetization curve of Pt/Co/Pd system can be obtained by only sweeping electrical current owing to the stability caused by the SOT. In addition, the magnetization direction can be reversibly controlled by simply changing the current polarity.

**Figure 4:**
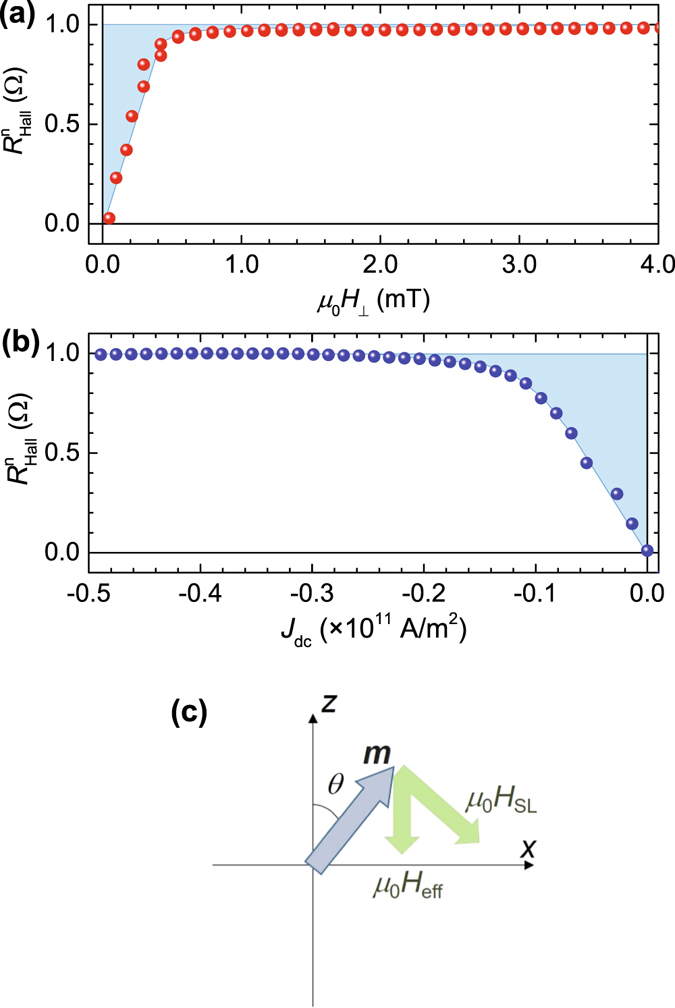
Quantitative determination of SOT effective field. (**a**) Normalized *R*_Hall_ as a function of *μ*_0_ *H*_z_ in range from 0 to +4.0 mT at 343 K. (**b**) Negative *J*_dc_ dependence of normalized *R*_Hall_ under *μ*_0_ *H*_x_ of +38 mT at 343 K. Zeeman energy gains by *μ*_0_ *H*_z_ and perpendicular component of the SOT effective field *μ*_0_ *H*_eff_ correspond to areas of coloured region indicated in a and b, respectively. The error bar, which is the standard deviation of two data points, is smaller than the symbols. (**c**) Schematic illustration displaying directions of the longitudinal effective field due to the Slonczewski-like torque *μ*_0_ *H*_SL_ and *μ*_0_ *H*_eff_ with respect to the magnetization direction *m*. Electrical current flows in +*x* direction.

The magnetization processes shown in Fig. 3(a) and (d) are caused by *μ*_0_ *H*_*z*_ and *μ*_0_ *H*_eff_, which is proportional to *J*_dc_, respectively. The current-induced magnetization process is expected to develop with the DW motion in the current direction, while the magnetic domains isotropically expands when the magnetization process is caused by the external field. Here, we focus on the gains of the Zeeman energy for each case and determine *μ*_0_ *H*_eff_, and consequently *μ*_0_ *H*_SL_, by comparing them. First, *E*_Z_ obtained by *μ*_0_ *H*_*z*_ (*E*_Z___*H*_) is calculated for the up magnetization case. Figure 4(a) shows the normalized *R*_Hall_ (*R*_Hall_^n^) as a function of *μ*_0_ *H*_*z*_ in the range from 0 to +4.0 mT. *E*_Z___*H*_ can be calculated using the following equation:

1 $${E}_{Z\_H}={M}_{{\rm{s}}}{\int }_{0}^{1}d{R}_{{\rm{Hall}}}^{{\rm{n}}}{\mu }_{0}{H}_{z},$$

where *M*_s_ is the saturation magnetization of the system. The integral term of (1) is defined by the coloured area of Fig. 4(a), and *E*_Z___*H*_/*M*_s_ is determined to be 0.180 mT. Subsequently, using the current*-*induced magnetization curve shown in Fig. 3(d), *E*_Z_ resulting from *μ*_0_ *H*_eff_ (*E*_Z___*J*_) is calculated. *R*_Hall_^n^ as a function of *J*_dc_ in the negative *J*_dc_ sweep, where the positive *μ*_0_ *H*_eff_ is applied to the system, is shown in Fig. 4(b). The small *J*_dc_ offset is corrected in this figure. Since *μ*_0_ *H*_eff_ is expected to be proportional to *J*_dc_, *E*_Z___*J*_ can be determined from the following:

2 $${E}_{Z\_J}=-{M}_{{\rm{s}}}{\int }_{0}^{1}d{R}_{{\rm{Hall}}}^{{\rm{n}}}\alpha {J}_{{\rm{dc}}},$$

where *α* is the constant value defined as *μ*_0_ *H*_eff_/*J*_dc_. The calculation of *E*_Z___*J*_/*M*_s_ is done in the same manner and is determined to be 0.662*α* × 10^10^ A/m^2^. The same calculations are performed for the down magnetization case. Assuming *E*_Z___*H*_/*M*_s_ = *E*_Z___*J*_/*M*_S_, *α* = 2.51 ± 0.01 mT/10^11^ A/m^2^ is finally obtained.

Next, we calculated *μ*_0_ *H*_SL_ (per 10^11^ A/m^2^) using *μ*_0_ *H*_eff_ determined above. Figure 4(c) shows a schematic where the direction of *μ*_0_ *H*_SL_ under the *x*-field is denoted. *μ*_0_ *H*_eff_ is the perpendicular component of *μ*_0_ *H*_SL_ and *θ* is the tilting angle of the magnetization from the *z*-axis. Thus, *μ*_0_ *H*_SL_ is expressed as *μ*_0_ *H*_eff_/sin*θ*. From the *x*-field dependence of the anomalous Hall resistance, *θ* at *μ*_0_ *H*_*x*_ = +38 mT is approximately 19° in our Pt/Co/Pd structure. Therefore, *μ*_0_ *H*_SL_ of 7.67 ± 0.04 mT/10^11^ A/m^2^ is obtained. This value shows good agreement with that obtained by the harmonic measurement in our Pt/Co/Pd system. *μ*_0_ *H*_eff_ was also calculated using a Langevin fit and the result is consistent with the direct Zeeman energy calculation (see Supplementary Information). The calculation results presented here indicate that the SOT effective field can be quantitatively determined from the current-induced magnetization curve.

**Figure 5:**
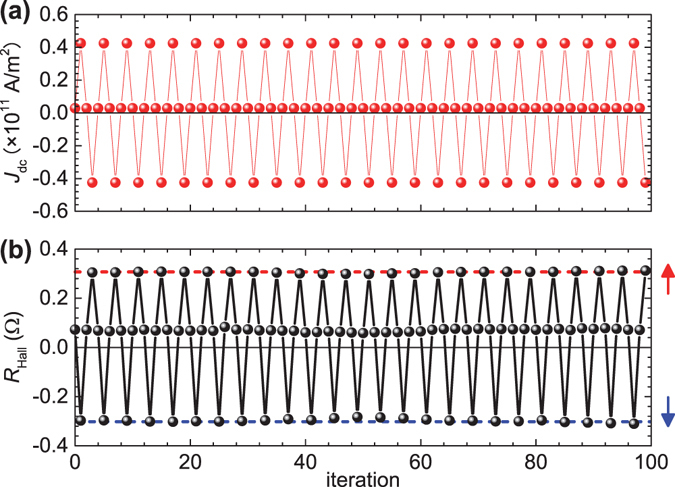
Alternative domain state switching. (**a**) Sequence of pulsed *J*_dc_ injection. *J*_dc_s of +0.4 × 10^11^, +2.8 × 10^9^, and −0.4 × 10^11^ A/m^2^ are repeatedly injected into the device. The pulse duration of each *J*_dc_ is 1.0 s. (**b**) Monitored *R*_Hall_ during alternative *J*_dc_ injection. Dashed lines indicate the saturation values of *R*_Hall_ in single domain state.

Finally, we demonstrate current-induced alternate switching between the MD and SD states. Figure 5(a) shows the sequence of *J*_dc_ injection into the device. A pulsed *J*_dc_ with three values of +0.4 × 10^11^, +2.7 × 10^9^, and −0.4 × 10^11^ A/m^2^ is injected in series to create the SD state with up and down magnetization. The duration of each *J*_dc_ pulse is 1.0 s, and a *μ*_0_ *H*_*x*_ of +38 mT is applied during the measurement. The *R*_Hall_ value is measured during the pulse injection, and the result is shown in Fig. 5(b). Maximum and minimum *R*_Hall_ values corresponding to the up and down SD states appear alternately with the injections of +0.4 and −0.4 × 10^11^ A/m^2^. At *J*_dc_ = 2.7 × 10^9^ A/m^2^, *R*_Hall_ always exhibits an intermediate value, that is, the MD state is restored. We checked that the current-induced SD state returns to the MD state within 1 ms after pulse off. This result indicates that an arbitrary switching of domain states between MD and SD can be achieved by injecting a current. Although in the present case, 1-ns-long pulses were used for the convenience of the measurement, sub-ns domain state switching is expected to be possible because the SOT induced magnetization switching occurs in this time scale^34^.

When the SOT effectively acts on the magnetization, the electrical current flowing in the system enhances the stability of the single domain state. In this study, owing to this domain stability, we show that the magnetization curve of the nonmagnetic/ferromagnetic metal structure can be obtained by sweeping electrical current, while it is conventionally obtained by sweeping external magnetic field. This effect would be marked in smaller size applicable to current IT devices because the SOT field is proportional to the current density flowing in the heavy metal layer. Anisotropy-wedged film^16^, an interlayer exchange coupled system^17^, and an antiferromagnet/ferromagnet layered structure^18^, where in-plane field free magnetization switching by the SOT was achieved, may also enable domain state switching without an in-plane field. In addition, this work offers a novel method to determine the SOT effective field from the current-induced magnetization curve.

## Methods

### Film deposition and device fabrication

Multilayered Ta (2.7 nm)/Pt (3.0)/Co(0.36)/Pd (0.8) film was deposited on a thermally oxidized Si substrate using rf sputtering, and a 0.5-nm-thick Ta layer was formed on the film as a cap. The base pressure of the sputter chamber was below 1.0 × 10^−6^ Pa, and Xe process gas was used for the deposition. The X-ray diffraction profile indicates that the Pt layer has an fcc (111) texture. The film was patterned into a Hall bar structure by photolithography and Ar ion milling. Cr (1.0)/Cu (100) electrodes were deposited by thermal evaporation and defined by a lift-off process using photolithography.

### Measurement setup

Measurements were performed using a prober system in which a vector magnetic field can be applied. The device temperature was controlled by a plate-shaped heater placed under the device and monitored using a Pt thermometer (Pt-100). A current source (Yokogawa 7651) and nano-voltmeter (Keithley 2182A) were used for anomalous Hall measurements.

## Electronic supplementary material

Supplementary Informations
