# Supplementary material for: Investigation of spin-orbit torque using current-induced magnetization curve
Source: Sci Rep. 2017 Apr 11;7:790. doi: 10.1038/s41598-017-00962-7 (PMC5429785; doi:10.1038/s41598-017-00962-7)
Supplement: Supplementary file 1 — Supplementary Informations [file 41598_2017_962_MOESM1_ESM.pdf]

Investigation of spin-orbit torque using current-induced magnetization curve

-Supplementary Information-

Tomohiro Koyama, Yicheng Guan and Daichi Chiba

7 figures are included in the SI.

Increase in device temperature as a function of injected current density

The increase in device temperature under the injection of an electric current (ΔT_J) is measured by investigating the dc current density J_{dc} dependence of the device resistance R. The result is shown in Fig. S1. Based on the R increase, ΔT_J is estimated using a temperature-resistance coefficient of 0.048 %/K, which was obtained in a device with a similar structure[1]. During the measurement, a constant $\mu_0 H_z$ of ~+10 mT is applied to prevent the formation of a multiple domain state that can additionally contribute to R as the domain wall resistance.

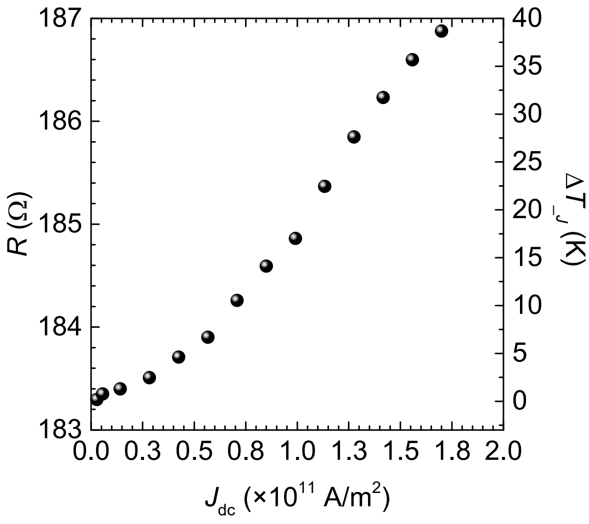

Figure S1 Injected dc current density J_{dc} dependence of the device resistance (R, left) and device temperature increase (ΔT_J, right).

Magnetization measurement

Figure S2a shows in-plane and perpendicular magnetization curves for the as-grown film obtained at 340 K. The curves are measured using a superconducting quantum interference device and a film size of 2 × 5 mm^2. The perpendicular magnetization curve shows a rapid saturation for a small $\mu_0 H_z$ of ~1 mT. In contrast, the magnetic susceptibility of the in-plane curve is quite small. These magnetization curves strongly indicate that the degradation of the squareness of the $\mu_0 H_z\_R_{Hall}$ curve at 343 K in our device (Fig. 3a) is not due to anisotropy switching from the perpendicular to the in-plane directions. The temperature T dependence of the perpendicular magnetization per area ($m_\perp / S$) is shown in Fig. S2b. From the $m_\perp / S - T$ curve, one can see that the Curie temperature of the film is approximately 370 K.

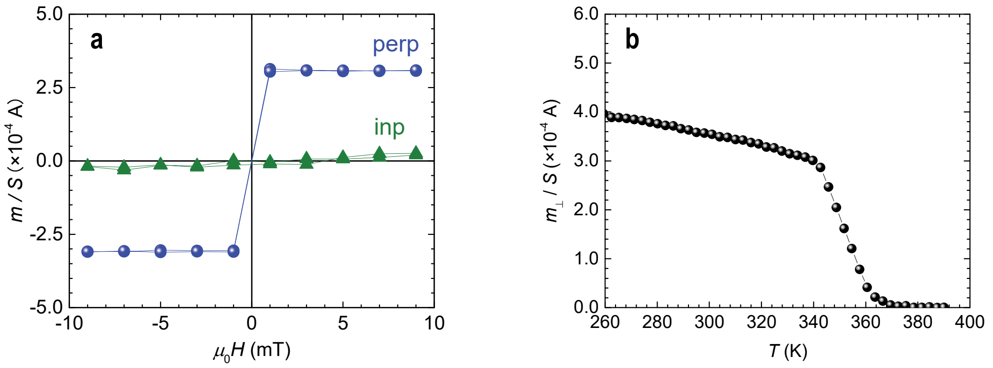

Fig. S2 (a) In-plane (triangles) and perpendicular (circles) magnetization curves for the as-grown

film at 340 K. (b) Temperature T dependence of the perpendicular magnetization per area ($m_{\perp}$ / S). T is increased at a rate of 3 K/min. A $\mu_0 H_z$ of ~ 0.5 mT is applied during the measurement.

Current density dependence of the coercivity and remanent Hall resistance

The coercivity $\mu_0 H_c$ and remanent Hall resistance R_{Hall}^r are investigated by changing J_{dc}. Figure S3a shows the $\mu_0 H_z$ dependence of the anomalous Hall resistance R_{Hall} for various J_{dc}. Clear hysteresis loops can be observed under J_{dc} of 2.8×10^9 A/m^2. $\mu_0 H_c$ decreases to 1.0 mT for $J_{dc} = 0.7\times10^{11}$ A/m^2. In both cases, the remanent value of R_{Hall} (R_{Hall}^r) is almost equal to the saturation value, indicating that the magnetic single domain (SD) state is stable at fields near zero. However, for $J_{dc} = 1.1\times10^{11}$ and 1.7×10^{11} A/m^2, $\mu_0 H_c$ and R_{Hall}^r become almost zero. Figure S3b shows R_{Hall}^r and $\mu_0 H_c$ as a function of J_{dc}. R_{Hall}^r is defined as the average of the absolute values of R_{Hall} at 0 T obtained in the positive and negative $\mu_0 H_z$ sweeps. One can see that $\mu_0 H_c$ monotonically decreases with increasing J_{dc} and almost becomes zero at $J_{dc} = 0.7\times10^{11}$ A/m^2, suggesting that the device temperature T_d gradually increases with J_{dc} owing to the Joule heating effect. While R_{Hall}^r is almost constant when $J_{dc} \leq 0.7\times10^{11}$ A/m^2, it suddenly decreases at $J_{dc} = 0.8\times10^{11}$ A/m^2 and stays near zero when J_{dc} is above this value. This result clearly indicates that the SD state cannot be maintained and that the formation of a multi-domain (MD) state occurs in the vicinity

of zero field by the injection of a J_{dc} larger than 0.8×10^{11} A/m^2, primarily owing to Joule heating.

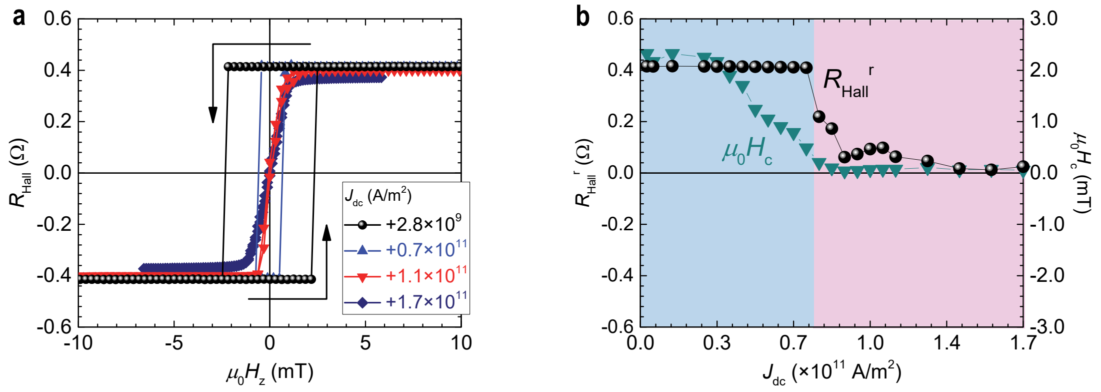

Figure S3 (a) Measurement of the anomalous Hall resistance R_{Hall} obtained by sweeping a perpendicular field μ_0H_z. The densities of the dc currents J_{dc} are $+2.8\times10^9$ (circle), $+0.7\times10^{11}$ (blue triangle), $+1.1\times10^{11}$ (red triangle), and $+1.7\times10^{11}$ A/m^2 (diamond). The μ_0H_z sweep rate was ~0.04 mT/s. The measurements were performed at a stage temperature of 304 K. The black arrows indicate the sweep direction. (b) Coercivity (μ_0H_c; triangle) and remanent R_{Hall} (R_{Hall}^r; circle) as a function of J_{dc}.

Initial magnetization curve obtained after high current injection

In Fig. S4a, we show the results of the additional J_{dc} sweeping measurement at 304 K. In this measurement, J_{dc} is first swept from $+2.8\times10^9$ to $+1.1\times10^{11}$ A/m^2 (1st sweep) and then reduced from $+1.1\times10^{11}$ to $+2.8\times10^9$ A/m^2 while R_{Hall} is monitored (2nd sweep). μ_0H_z is set to ~0 T. As shown in this figure, R_{Hall} does not return to its initial value during

the 2nd sweep. After this measurement, a μ_0H_z sweep is performed at a J_{dc} of $+2.8\times10^9$ A/m^2 (Fig. S4b). R_{Hall} gradually increases from the intermediate value (~0.1 Ω) to the saturation value as μ_0H_z increases from 0 to ~1 mT, corresponding to the initial magnetization curve (1st sweep). After the saturation, a clear hysteresis loop of R_{Hall} is confirmed (2nd sweep). A similar result is obtained when the J_{dc} sweep measurement is carried out under a *y*-field of 38 mT. These results provide evidence that the MD formation occurs owing to the application of a high J_{dc}.

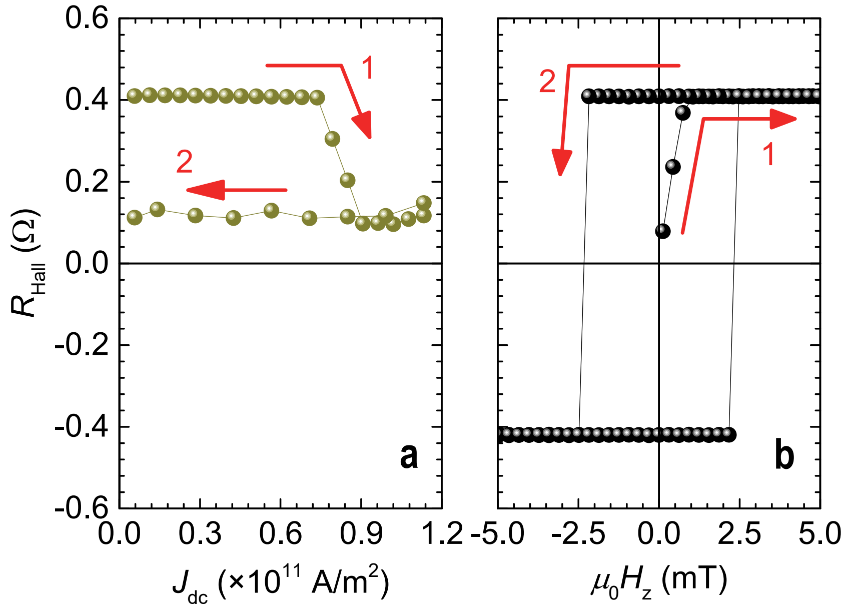

Figure S4 (a) Results of R_{Hall} monitoring with sweeping J_{dc}. First, J_{dc} is changed from $+2.8\times10^9$ to $+1.1\times10^{11}$ A/m^2 (sweep 1); it is then reduced to $+2.8\times10^9$ A/m^2 (sweep 2). (b) Hall measurements performed after the measurement shown in (a). μ_0H_z is initially increased from 0 to +5 mT (sweep 1). Then, the hysteresis measurement is performed (sweep 2).

Harmonic Hall measurement

To determine the magnitude of the Slonczewski-like and field-like torques, the harmonic Hall measurement[2] is performed in a reference Pd/Co/Pt structure. The structure of the reference is Ta(2.7 nm) / Pt (3.0) / Co(0.40) / Pd (0.8) / Ta (0.5). The detail of the technic is described in ref. S2. AC current with the frequency ω of 1 kHz is injected to the device. The amplitude of the current is 2.0 mA. The in-phase first harmonic anomalous Hall voltage (V_ω) and out-of-phase second harmonic one ($V_{2\omega}$) are measured using the lock-in amplifier with sweeping in-plane field parallel ($\mu_0 H_x$) and perpendicular ($\mu_0 H_y$) to the current. Figure S5a and b show $\mu_0 H_x$ dependences of V_ω and $V_{2\omega}$, respectively. The longitudinal effective field due to the Slonczewski-like torque $\mu_0 H_{SL}$ is expressed as;

$$\mu_0 H_{SL} = -2\left(\frac{dV_{2\omega}}{d\mu_0 H_x}\right)/\left(\frac{d^2 V_\omega}{d\mu_0 H_x^2}\right). \quad (1)$$

Using (1), we obtain $\mu_0 H_{SL} = 6.55 \pm 0.20$ mT / 10^{11} Am^{-2}.

$\mu_0 H_y$ dependences of V_ω and $V_{2\omega}$ are shown in fig. S5c and d. Similarly, the transverse effective field caused by the field-like torque $\mu_0 H_{FL}$ is expressed as;

$$\mu_0 H_{FL} = -2\left(\frac{dV_{2\omega}}{d\mu_0 H_y}\right)\Big/\left(\frac{d^2 V_\omega}{d\mu_0 H_y^2}\right). \quad (2)$$

Thus, the magnitude of $\mu_0 H_{FL}$ is determined to be 1.57 ± 0.13 mT / 10^{11} Am^{-2}.

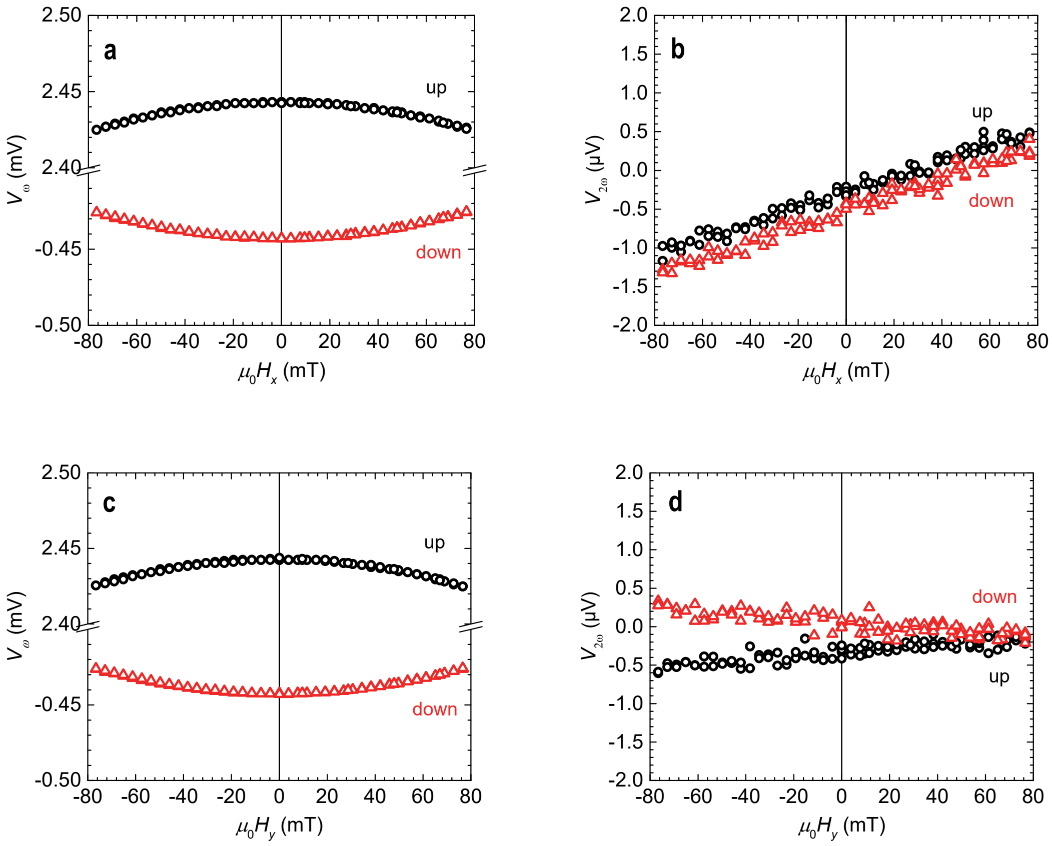

Figure S5 (a,b) First (V_ω) and second ($V_{2\omega}$) harmonic anomalous Hall voltages as a function of in-plane field parallel to the current (μ_0H_x). The result for up (down) magnetization configuration is indicated by the black circle (red triangle). (c,d) V_ω and $V_{2\omega}$ as a function of in-plane field perpendicular to the current (μ_0H_y)

Domain structure

Observations of the magnetic domain structure of the as-grown film are carried out using polar magneto-optical Kerr effect (MOKE) at 345 K. Figures S6a – c show MOKE images acquired at μ_0H_z values of -2, ~0, and +2 mT, respectively. To enhance the contrast, each

image is subtracted from the image acquired under high μ_0H_z. When a μ_0H_z of +2 (-2) mT is applied, no domain can be observed because the film is in the single domain state with the up (down) magnetization. On the other hand, a stripe-like domain pattern can be seen in the vicinity of zero field. The Hall measurement performed at 343 K shows a R_{Hall}^r of almost zero, as displayed in Fig. 4a. These results indicate that the zero R_{Hall}^r is due to multiple domain formation at 343 K.

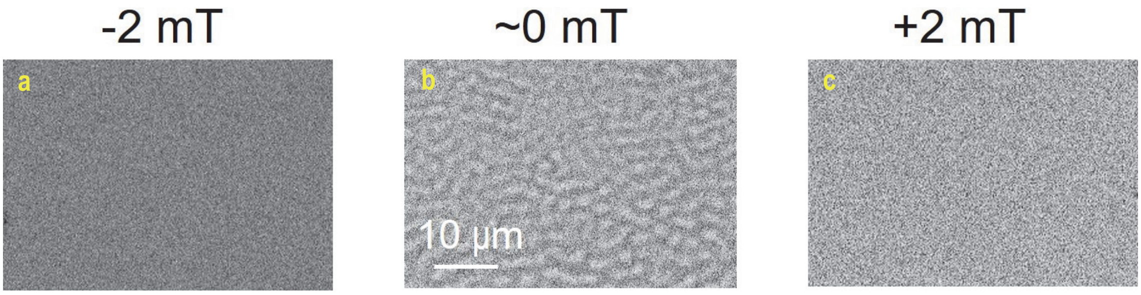

Figure S6 MOKE images for μ_0H_z of (a) -2, (b) ~0, (c) +2 mT, obtained at 345 K. The observation area is 35 × 52 μm^2.

Effective field determination using the Langevin fit

The reversible magnetization process without hysteresis as Fig. 3 can be reproduced by the Langevin function[3]. Here, we show the result of the Langevin fit to the Fig. R3 data and μ_0H_{eff} obtained with this method. When a ferromagnetic system has uniaxial anisotropy, the normalized Langevin function is known to be simple formula: $\tanh(m\mu_0H / k_BT)$; where m is magnetic moment, k_B the Boltzman constant and T the temperature.

Since the magnetization of the present Pt/Co/Pd system points only up or down due to the strong perpendicular magnetic anisotropy PMA, we adopted the simplified Langevin function for the fit. Fig. 3(d) data was fitted by the following function; $R_s*\tanh(m(\alpha J_{dc} - \beta) / k_B T)$, where Rs is the saturation value of R_{Hall}, $\alpha = \mu_0 H_{eff} / J_{dc}$ and β is the offset. The value of m was determined from the data in Fig. 3(a). The fitting result is shown in Fig. S7 and α is determined to be 2.34 ± 0.11 mT / 10^{11} Am^{-2}. In the same way as the direct calculation shown in the main text, $\mu_0 H_{SL}$ (per unit mA) is determined to be 7.13 ± 0.38 mT / 10^{11} Am^{-2}. This value is consistent with that obtained by the direct Zeeman energy calculation.

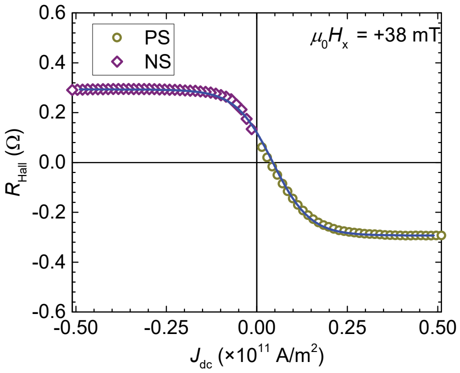

Figure S7 Results of the Langevin fit for current-induced magnetization curve (Fig. 3(d) in the main text). The solid line shows the fit.

Reference

S1. Koyama, T. & Chiba, D. Determination of effective field induced by spin-orbit torque using magnetic domain wall creep in Pt/Co structure. *Phys. Rev. B* **92**, 220402(R) (2015).

S2. Kim, J. *et al.* Layer thickness dependence of the current-induced effective field vector in Ta/CoFeB/MgO. *Nature Materials* **12**, 240-245 (2013).

S3. Hauser, H., Melikhov, Y. & Jiles, D. C. Examination of the Equivalence of Ferromagnetic Hysterisis Models Describing the Dependence of Magnetization on Magnetic Field and Stress. *IEEE. Trans. Magn.* **45**, 1940 (2009).
